# A Novel Antimicrobial Peptide, Dermaseptin-SS1, with Anti-Proliferative Activity, Isolated from the Skin Secretion of *Phyllomedusa tarsius*

**DOI:** 10.3390/molecules28186558

**Published:** 2023-09-11

**Authors:** Xiaonan Ma, Yuping Chen, Anmei Shu, Yangyang Jiang, Xiaoling Chen, Chengbang Ma, Mei Zhou, Tao Wang, Tianbao Chen, Chris Shaw, Lei Wang

**Affiliations:** 1 Natural Drug Discovery Group, School of Pharmacy, Queen’s University Belfast, Belfast BT9 7BL, UK; xma05@qub.ac.uk (X.M.); x.chen@qub.ac.uk (X.C.); c.ma@qub.ac.uk (C.M.); m.zhou@qub.ac.uk (M.Z.); t.chen@qub.ac.uk (T.C.); chris.shaw@qub.ac.uk (C.S.); l.wang@qub.ac.uk (L.W.); 2 Department of Basic Medical Science, Jiangsu Vocational College of Medicine, Yancheng 224005, China; 12064@jsmc.edu.cn (Y.C.); 12230@jsmc.edu.cn (A.S.)

**Keywords:** antimicrobial peptide, dermaseptin, molecular cloning, analogue design

## Abstract

The emergence of multidrug-resistant bacteria has severely increased the burden on the global health system, and such pathogenic infections are considered a great threat to human well-being. Antimicrobial peptides, due to their potent antimicrobial activity and low possibility of inducing resistance, are increasingly attracting great interest. Herein, a novel dermaseptin peptide, named Dermaseptin-SS1 (SS1), was identified from a skin-secretion-derived cDNA library of the South/Central American tarsier leaf frog, *Phyllomedusa tarsius,* using a ‘shotgun’ cloning strategy. The chemically synthesized peptide SS1 was found to be broadly effective against Gram-negative bacteria with low haemolytic activity in vitro. A designed synthetic analogue of SS1, named peptide 14V5K, showed lower salt sensitivity and more rapid bacteria killing compared to SS1. Both peptides employed a membrane-targeting mechanism to kill *Escherichia coli*. The antiproliferative activity of SS1 and its analogues against lung cancer cell lines was found to be significant.

## 1. Introduction

In February 2017, the World Health Organization (WHO) released its inaugural inventory of antibiotic-resistant “priority pathogens”. This comprehensive compilation encompassed 12 bacterial families that provide the most significant peril for human well-being. The inventory emphasized the menace caused by Gram-negative bacteria that demonstrate resistance to multiple antibiotics. The mentioned bacteria possess the inherent capacity to develop novel mechanisms of resistance against treatments while being capable of transferring genetic material that facilitates the acquisition of drug resistance by other microbes. The most noteworthy cohort encompassed multidrug-resistant bacteria, which present a specific risk to healthcare facilities, long-term care facilities, and individuals requiring medical interventions, such as mechanical ventilation and intravenous catheterization. These include *Acinetobacter*, *Pseudomonas*, and *Enterobacteriaceae* (*Klebsiella*, *E. coli*, *Serratia*, and *Proteus*). These microorganisms can potentially induce severe and frequently fatal infections, such as bacteraemia and pneumonia [1]. Bacteria are resistant to various antibiotics, such as carbapenems and third-generation cephalosporins, which are considered the most effective treatment options for combating bacterial resistance to multiple drugs. Hence, it is imperative to uncover new antimicrobial drugs. Antimicrobial peptides have proven effective in eradicating bacteria while exhibiting a limited propensity for inducing drug resistance.

Dermaseptins represent a group of peptides extracted from the skin secretions of frogs belonging to the *Hylidae* family [2]. The two main attributes of dermaseptins are their antimicrobial and antitumor effects. Many peptides exhibit lethality towards bacteria that lack cell walls and both Gram-negative and Gram-positive bacteria, fungi, and yeasts, but they have shown lower toxicity towards mammalian cells [3,4,5]. The antibacterial activity of these substances is attributed to their ability to bind to the plasma membrane of bacteria, inducing temporary wormholes or membrane disruptions [6]. Considering the escalating resistance exhibited by bacteria towards conventional antibiotics, there is a significant need for novel antibacterial medications [7]. Similarly, new antitumor therapies are needed in parts of the world where cancer is increasingly emerging as the primary cause of mortality, as conventional therapies exhibit non-selective cytotoxicity and are susceptible to the development of resistance as a result of the microevolutionary processes in tumor cells. Members of the dermaseptin family additionally demonstrate specific cytotoxic and antiproliferative effects on human tumor cell lines and retain spermicidal and antiprotozoal characteristics [8]. According to the APD3 Database (https://aps.unmc.edu/ (accessed on 1 August 2023)), peptides from the dermaseptin family display significant antiproliferative activity against lung cancer cell lines. Dermaseptin-PS3 from *Phyllomedusa sauvagii* has shown antimicrobial activity against *E. coli* and *C. albicans* and antiproliferative activity against lung cancer cell lines [9]. Both dermaseptin L1 and phylloseptin L1, which were obtained from the skin secretions of the lemur leaf frog, *Agalychnis lemur* (*Phyllomedusinae*), exhibited cytotoxic effects on hepatocarcinoma HepG2 cells following stimulation with norepinephrine [10].

## 2. Results

### 2.1. The ‘Shotgun’ Cloning of Dermaseptin-SS1 Precursor-Encoding cDNA from the Skin Secretion of Phyllomedusa Tarsius

**Figure 1:**
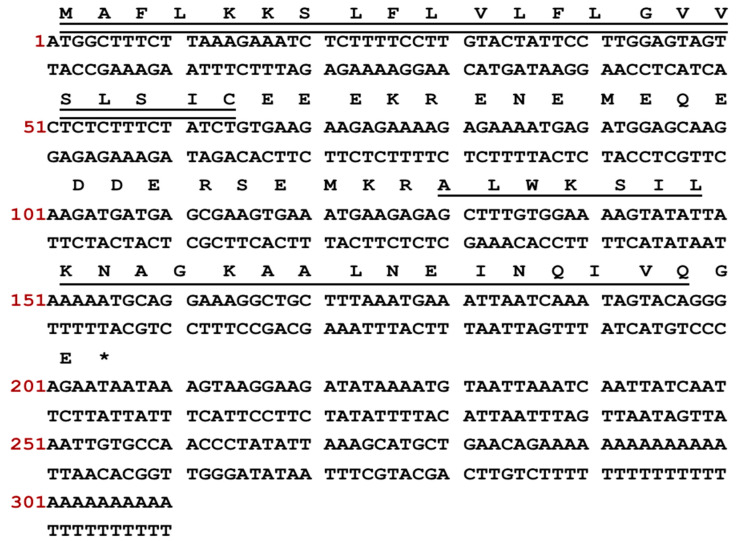
The ORF of the cDNA and nucleotide sequence that encoded the biosynthetic precursor of dermaseptin-SS1 were translated. The N-terminal signal peptide sequence is denoted by double underlining, whereas the mature peptide sequence is indicated using single underlining. The stop codon is marked by the symbol “*”, signifying the termination of protein synthesis.

The complementary DNA (cDNA) encoding the precursor of the newly discovered peptide was cloned from the cDNA library derived from the skin secretion of *Phyllomedusa tarsius*. The nucleotide sequence is depicted in Figure 1, and the precursor’s open reading frame (ORF) comprised 68 amino acid residues. Five discrete domains were present as follows: a signal peptide region, an acidic spacer peptide domain, a processing protease cleavage site, a mature peptide region, and a glycine residue amide donor. A signal peptide domain consisting of 22 residues was identified at the N-terminus of the reading frame. An acidic spacer domain consisting of 19 residues was observed preceding the mature peptide sequence. The letters ‘KR’ (Lys-Arg) indicated the presence of a protease cleavage site. The mature peptide sequence was found to comprise 23 amino acids. The occurrence of an extension sequence ‘GE’ (-Gly-Glu-) was observed, wherein the glycine residue served as the donor for the carboxyl (COOH)-terminal amidation of the mature peptide. The nucleotide sequence of the Dermaseptin-SS1 precursor was archived in GenBank (accession number: OR365763).

**Figure 2:**
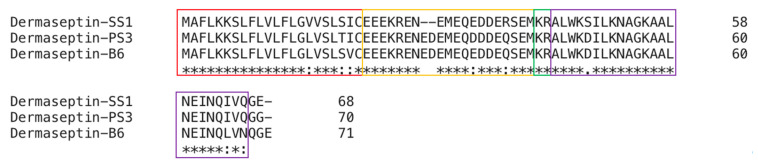
The alignment of full-length nucleotide sequences of cloned encoding precursors of SS1, Dermaseptin-PS3, and Dermaseptin-B6. The sequences in the red box are signal peptides, those in the yellow box are acidic spacer peptide regions, and those in the purple box are mature peptides. The identical amino acids are represented by “*”. The presence of a colon “:” denotes conservation among groups of amino acids that possess highly similar properties. The presence of a colon “.” denotes conservation among groups that exhibit weakly similar properties.

The peptides dermaseptin-PS3 and dermaseptin-B6 related to dermaseptin-SS1 (SS1) were found in the NCBI-BLAST programme. Dermaseptin-PS3 from the skin secretion of *Phyllomedusa sauvagii* was shown to have a 96% sequence identity with SS1, whereas dermaseptin-B6 from the skin secretion of *Phyllomedusa bicolor* displayed a 91% sequence identity with SS1. The full-length nucleotide sequences of the three AMPs were compared using the Clustal-Omega programme, and the results are displayed in Figure 2.

### 2.2. Physicochemical Properties and Structural Modification of SS1

The mature amino acid sequences of peptides are presented in Table 1. There were three steps in the modification of SS1. The analogues Lack14 (L14), 14V, and 14G were modified first by increasing hydrophobic characteristics and decreasing helicity. The second step used L14 as the template and obtained the analogue L14+10,13V (L2V) with a higher hydrophobic property. The last design was 14V5K and 14VL23 for increasing the net charge and hydrophobic properties using 14V as the model. Table 2 displays the physicochemical properties predicted using Heliquest. Hydrophobicity, hydrophobic moment, and net charge were related to the side chains of amino acids.

### 2.3. Secondary Structure Analysis of SS1 and Its Analogues

**Figure 3:**
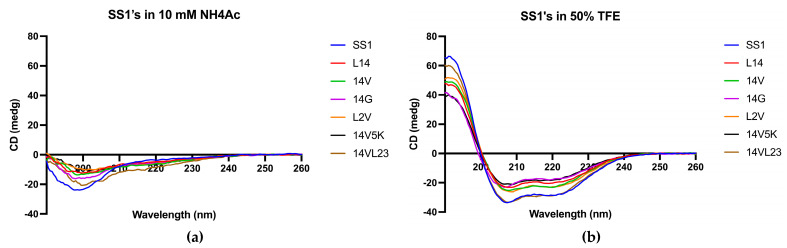
The CD spectra of SS1 and its analogues were obtained in two different environments: (**a**) a 10 mM NH_4_Ac buffer, encompassing an aqueous environment, and (**b**) a 50% TFE solution, simulating the hydrophobic conditions found in the microbial membrane. Peptides demonstrated α-helical conformations in solutions that mimicked membranes due to the presence of distinctive positive peaks at 193 nm and negative peaks at 208 nm and 222 nm. Conversely, in aqueous environments, they adopted a distinct random-coil conformation.

Following the synthesis process, the peptides were purified using RP-HPLC, and their molecular weights were subsequently verified using MALDI-TOF mass spectrometry (Table S1, Figure S1). The retention time (RT) of SS1 and its analogues ranged from 49 to 63 min, and the purity of the peptides reached 99% (Figure S2). Figure 3 displays the circular dichroism (CD) spectra of peptides in two distinct solvents, namely a solution containing 10 mM ammonium acetate (NH4Ac), representing an aqueous milieu, and a membrane-mimetic solution composed of 50% (*v*/*v*) trifluoroethanol (TFE) in NH4Ac.

### 2.4. Anti-Planktonic Microorganism Activity Selection of SS1 and Its Analogues

The antimicrobial properties of SS1 and its analogues are presented in Table 3. The minimal inhibitory concentration (MIC) and minimal bactericidal concentration (MBC) of the peptides were measured against Gram-positive bacteria, Gram-negative bacteria, and yeast.

### 2.5. Haemolysis Activity of SS1 and Its Analogues

Concerning the haemolytic activity towards horse erythrocytes, SS1, and its analogues exhibited the same HC_50_ values, as shown in Table 4. 14V5K showed an obvious advantage in the therapeutic index compared to other peptides. Based on these results, further experiments on the antimicrobial activities and mechanisms of 14V5K will be conducted.

### 2.6. TI Analysis of SS1 and 14V5K against Antibiotic-Resistant E. coli

In terms of clinical isolates and drug-resistant strains, 14V5K exhibited better activities against most tested strains than SS1, as shown in Table 5. The TI values from Table 6 show that 14V5K had better therapeutic potential against these types of *E. coli* than SS1.

### 2.7. Salt Sensitivity Activity Research of 14V5K

An assessment was conducted to determine the sensitivities of 14V5K based on the MIC and MBC values of peptides under physiological conditions. The results are shown in Table 7. For 14V5K, the treatments with different ions slightly affected its antimicrobial performance against the test bacteria.

### 2.8. Time-Killing Kinetic Studies on 14V5K

**Figure 4:**
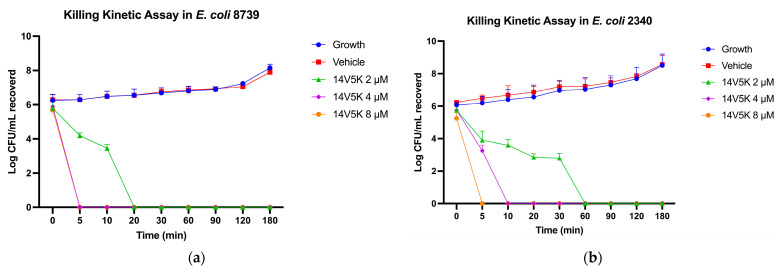
The kinetic time-killing curves of 14V5K (**a**,**b**) were assessed. Exponential-phase *E. coli* 8739 and *E. coli* 2340 were subjected to various concentrations ranging from MIC to 4-fold MIC (2–8 μM). The error bar represents the standard error of the mean (SEM) calculated from three replicates obtained from three separate experiments.

The Gram-negative bacterial strains, *E. coli* ATCC CRM 8739 and *E. coli* ATCC BAA 2340, were selected to investigate and compare the bactericidal kinetics of peptides 14V5K. It killed bacteria within less than 60 min at MIC concentration (Figure 4). However, at concentrations of two-fold MIC and four-fold MIC, all tested peptides eradicated bacteria within 10 min.

### 2.9. LPS-Binding Study of 14V5K

**Figure 5:**
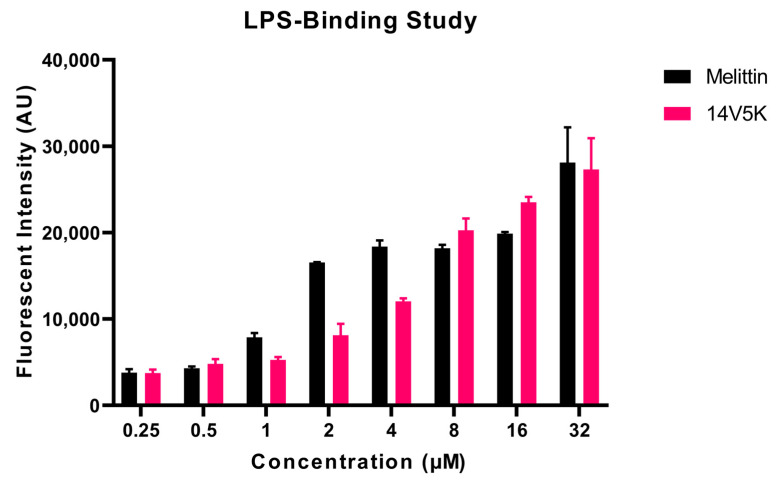
The binding affinity of the peptides to LPS was identified using the BC fluorescent dye displacement assay. The fluctuations in fluorescent values were observed at an λ_excitation_ = 590 nm and λ_emission_ = 645 nm. The data presented in this study represent the means and standard deviations of three separate trials.

The ability of the substances to interact with lipopolysaccharide (LPS) was assessed using a fluorescence-based displacement assay. The results indicated that 14V5K exhibited a concentration-dependent binding ability to LPS, similar to that of SS1 and melittin (Figure 5, Figure S4). The fluorescent intensity of 14V5K at concentrations of 1 μM to 4 μM was obviously lower than that of melittin.

### 2.10. Outer Membrane Permeability Study of 14V5K

**Figure 6:**
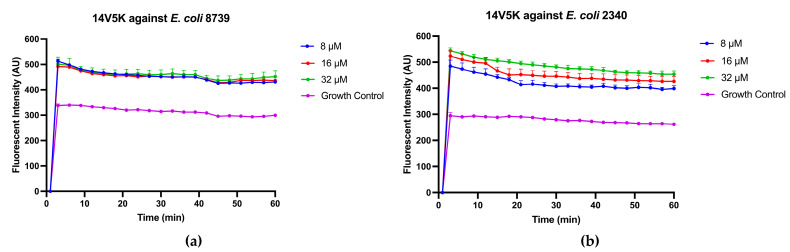
The outer membrane permeabilization of 14V5K. Exponential-phase *E. coli* 8739 (**a**) and *E. coli* 2340 (**b**) cells were treated with MIC to 4-fold MIC concentrations (8–32 μM). The SEMs are visually represented by the error bars displayed in the graphs.

To study the effects of 14V5K on the membrane integrity, two *E. coli* strains, *E. coli* ATCC CRM 8739 and *E. coli* ATCC BAA 2340, were selected. Peptide 14V5K initially induced changes in the permeability of the outer membrane, a trend similar to that of SS1 and melittin (Figure 6, Figure S5). 

### 2.11. Inner Membrane Permeability Study of 14V5K

**Figure 7:**
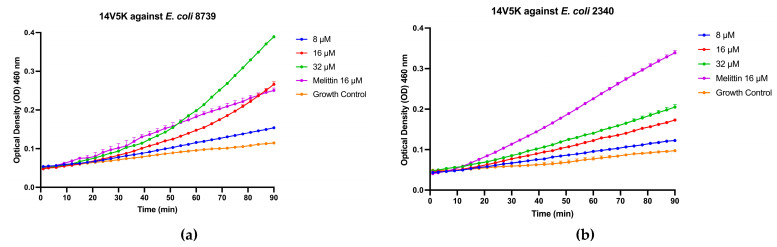
Inner membrane permeabilization of *E. coli* 8739 (**a**) and *E. coli* 2340 (**b**) after treatment with 14V5K. The spectroscopic measurement of the hydrolysis of ONPG, produced by the release of cytoplasmic β-galactosidase from *E. coli*, was measured at 460 nm over a period of 90 min.

The peptide-induced intracellular membrane permeabilization of *E. coli* ATCC CRM 8739 and *E. coli* ATCC BAA 2340 was assessed using the ONPG assay. The 14V5K-induced membrane permeabilization of *E. coli* obviously increased, as shown in Figure 7a,b, suggesting an augmented synthesis of *o*-nitrophenol by ONPG hydrolysis. However, the parent peptide SS1 produced almost no absorbance increase, as shown in Figure S6.

### 2.12. Membrane Potential Study of 14V5K

**Figure 8:**
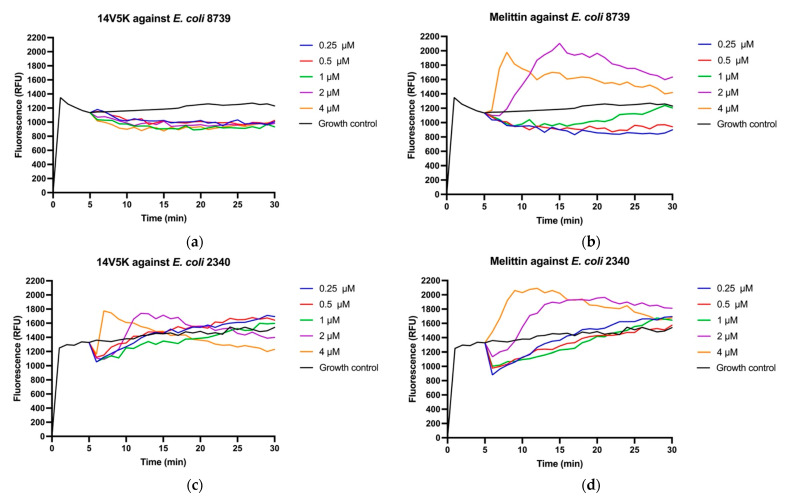
Cytoplasmic membrane depolarization of *E. coli* 8739 (**a**,**b**) and *E. coli* 2340 (**c**,**d**) was observed by employing the membrane potential dye, diSC_3_-5. 14V5K and melittin were measured at concentrations spanning from 0.5 μM to 4 μM.

The use of the fluorescent dye diSC3-5 was employed to investigate the depolarizing effects of the peptides on membrane potential. To account for the fluorescence enhancement caused by the peptides, the temporal variation in fluorescent values was monitored over 30 min (Figure 8). 14V5K displayed a low disruption effect on membrane potential against *E. coli* 8739, whereas it showed a similar tendency of fluorescence as SS1 and melittin against *E. coli* 2340 (Figure S7).

### 2.13. Swimming Motility Activity of 14V5K

**Figure 9:**
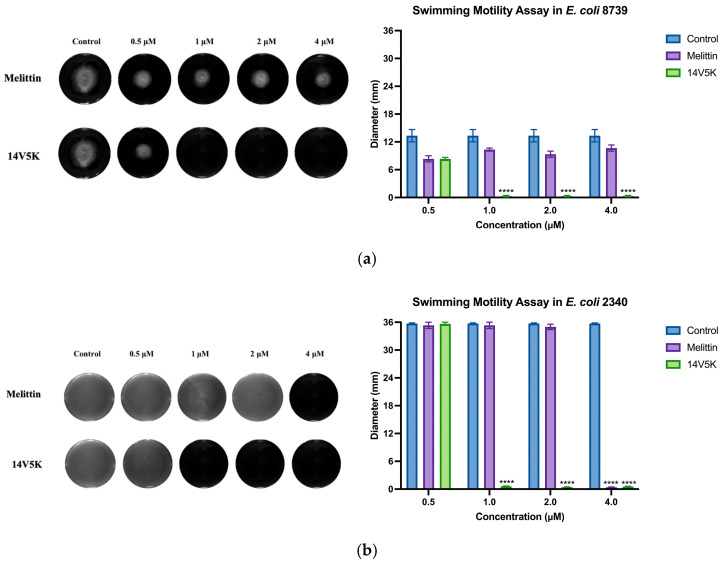
The histogram presented in this study displays the swimming motility data of the *E. coli* strains 8739 (**a**) and 2340 (**b**). The values depicted in the histogram represent the means ± standard deviations, which were calculated based on three separate experiments. The statistical significance (****) was established by employing a threshold of a *p*-value lower than 0.0001, associated with the bacterial swimming diameter observed in the control group that did not receive any antimicrobial treatment.

The effects of the peptide on the swimming motility of *E. coli* 8739 and 2340 were subsequently assessed using low-viscosity swim plates (Figure 9). The peptide 14V5K exhibited a concentration-dependent repression of bacterial swimming motility, with its effects surpassing those of SS1 and melittin at equivalent concentrations (Figure S8).

### 2.14. Antiproliferative Activity Study of SS1 and Its Analogues

The human lung cancer cell lines, H838 and H460, and the human keratinocyte cell line, HaCat, were used to measure the antiproliferative activities of SS1 and its analogues (Table 8). All peptides exhibited significant effects in both cancer cell lines, with L2V showing particularly pronounced effects. However, they also displayed noticeable antiproliferative effects against the normal HaCat cells concurrently. The selective index (SI) of the peptides is shown in Table 9.

## 3. Discussion

This study reported a newly discovered antimicrobial peptide, Dermaseptin-SS1, from the skin secretion of *Phyllomedusa tarsius*. The expected secondary structure of SS1 was identified as an amphipathic α-helix configuration, which was subsequently confirmed using CD spectrum analysis. To assess the potential efficacy of SS1 against drug-resistant bacteria, an in vitro investigation was conducted to examine its anti-planktonic microorganism activity. The antibacterial mechanism was also studied.

### 3.1. Modification of SS1

There were three main steps of structural modification, according to the evidence of the antimicrobial results. The predicted information from HeliQuest referred to the design of analogues (Figure 2). The first modification step was designed to increase hydrophobic characteristics and decrease helicity. Since SS1 exhibited some levels of functional activity with fragmented hydrophobic residues, our initial focus in modifying SS1 was to enhance its hydrophobic properties. L14 was designed to remove Alanine (Log P: 3.54) at position 14 to obviously increase the hydrophobic moment (Figure 2). The secondary structure was changed, and the predicted helical wheel plot is shown in Figure S9b. Position 14 continued to be used for modification. Peptide 14V had an Alanine to Valine (Log P: 4.42) substitution at position 14 to increase both hydrophobicity and hydrophobic moment. Valine possesses a moderate level of hydrophobicity, and the choice of valine was made to prevent an increase in cytoxicity. According to previous reports, most dermaseptin peptides have a specific hinge structure because of the amino acid Glycine (Log P: 3.20) [2,11]. Meanwhile, SS1 exhibited antimicrobial activity even in the presence of a glycine residue in its amino acid sequence. The substitution of alanine with glycine at position 14 in peptide 14G was intended to reduce helicity and potentially enhance the flexibility of the peptide’s structure, favoring the formation of a hinge-like structure. As expected, this modification indeed had a significant impact on the secondary structure of the peptide. Comparing the antimicrobial activity of the three analogues, L14 exhibited the most potent antimicrobial effect, suggesting that enhancing the hydrophobic moment could improve the activity to some extent. Since enhancing hydrophobicity significantly improved L14’s antibacterial performance, the second step of the modification process aimed to further enhance its hydrophobic properties. Based on the structure of L14, a further increase in the hydrophobic moment was achieved by changing Alanine to Valine at both positions 10 and 13. This analogue was named L2V. The antimicrobial activities of L2V greatly increased compared to those of L2, implying that an appropriate increase in hydrophobicity may be favorable for AMPs’ modification. Considering the antimicrobial properties of these peptides, since 14V showed the best effects among all five of the above peptides, it was used as a template for further modification. In the third step, the modification focused on increasing the net charge and further improving hydrophobicity. The analogue 14V5K was designed by substituting serine (Log P: 2.66) with lysine at position 5. This substitution was chosen because the serine side chain is neutral and replacing it at position 5 may enhance the amphipathicity of the helical domain. 14VL23 was designed by deleting Glutamine (Log P: 3.19) at position 23 as the C-terminal ended with a hydrophobic amino acid, which might increase hydrophobic properties [12,13]. However, the remarkably decreased antimicrobial activities of 14V23L suggested that Glutamine at the 23rd position may play an important role in the antimicrobial activities of this peptide. 

The findings of the CD spectra indicated that SS1 and its analogues exhibited α-helical conformations in solutions that mimic the properties of biological membranes, as shown in Figure 3. It was evident from the presence of characteristic positive and negative peaks in their spectroscopic data. However, these molecules exhibited distinct random-coil conformations in an aqueous environment. The obtained results illustrated a significant level of resemblance to the anticipated outcomes.

### 3.2. Anti-planktonic Microorganism Activity

The antimicrobial efficacy of SS1 and similar compounds was found to be more pronounced against Gram-negative bacteria than Gram-positive bacteria, particularly those resistant to conventional drugs, as shown in Table 3. However, their effectiveness against yeast was found to be limited. The peptide designated as 14V5K displayed superior antimicrobial efficacy against a broad spectrum of microorganisms compared to other peptides. However, compared to SS1, 14VL23 solely demonstrated antimicrobial activity against *E. coli* 8739 and *K. pneumoniae* 43816. In a comparison of the TI values of SS1 and its analogues in Table 4, 14V5K showed an obvious antimicrobial effect. Therefore, SS1 and 14V5K were selected to measure the future antimicrobial activities and mechanisms.

### 3.3. Antibiotic-resistant E. coli

As the peptides showed significant antimicrobial activity against *E. coli* 8739, additional strains of drug-resistant *E. coli* were used in this study. These were as follows: (i) *E. coli* 2340: KPC strain panel, bl_KPC_+/bla_NDM_-; (ii) *E. coli* 2469: NDM-1 strain panel, bla_NDM_+/bla_KPC_-; (iii) *E. coli* 2471: NDM-1 strain panel, bla_NDM_+/bla_KPC_-; (iv) *E. coli* 13846: colistin-resistant and MCR-1 positive. *E. coli* 2340, 2469, and 2471 belonged to carbapenem-resistant *Enterobacteriaceae* (CRE) [14]. Carbapenem belongs to the category of β-lactam antibiotics, functional by impeding the synthesis of peptidoglycan [15]. The bacteria resistant to carbapenem can be attributed to the production of carbapenemase, often accompanied by mutations in porins [16]. CRE has three major categories, including KPC, NDM, and OXA-48. *Klebsiella pneumoniae*. Carbapenemase (KPC) hydrolysed all β-lactam agents encoded by the plasmid-associated gene blaKPC, which may be difficult to detect using higher breakpoints [17]. The New Delhi Metallo-β-lactamase (NDM) was encoded by the plasmid-associated gene bla_NDM_ [18]. However, *E. coli* 13,846 was a colistin-resistant bacterium that was MCR-1 positive and harboured multiple other resistance genes. The MCR-1 protein, found on the surface of *E. coli* 13,846, has been reported to protect against the action of hydrophobic antibiotics, such as colistin [19]. This may theoretically explain the varying MICs listed in Table 5.

14V5K showed significant superiority in salt sensitivity against both bacteria, as shown in Table 7. However, for SS1, each type of salt could inactivate the antimicrobial activity against *S. aureus* ATCC CRM 6538, whereas salts had less influence on activity against *E. coli* ATCC CRM 8739, as shown in Table S2. The results were analysed from the perspective of the peptide properties. On the one hand, 14V5K had more net positive charges than SS1, so it was more competitive than most ions. The combination of the negatively charged LPS and peptide 14V5K produced a more vital electrostatic interaction, which was one of the factors of the membrane-targeting mechanism. On the other hand, 14V5K had a higher hydrophobic moment than SS1, and with this elevated hydrophobicity, the peptide readily accumulated on the phospholipid bilayers of the cell membrane. Therefore, 14V5K had a low salt sensitivity. Meanwhile, SS1 displayed long-duration killing for about 2 h (Figure S3), whereas 14V5K slightly decreased the killing time against bacteria because of the extra net charge and hydrophobic properties.

### 3.4. Antimicrobial Mechanism Analysis

The initial reaction between the cationic peptide and the cell membrane involves the negatively charged components of the membrane. A fluorescence-based displacement assay was conducted to assess the specificity of the binding of the peptides to LPS. The antimicrobial peptide SS1 exhibited similar levels of efficiency in displacing LPS as melittin, a well-known membrane-targeting peptide that can rapidly induce pore formation in bacterial cell membranes, leading to cytoplasmic leakage [20]. Once peptides aggregate on the surface of bacteria and reach a certain concentration threshold, they can penetrate the cell wall and destabilize the cell membrane [21]. However, 14V5K exhibited weaker LPS-binding ability at MIC concentrations. Therefore, this study investigated the immediate effects of peptides on both the external and internal membranes of *E. coli*. The alterations in the fluorescence of NPN and the absorbance of ONPG, as observed in the presence of SS1 and 14V5K, verified that peptides may penetrate the bacterial membrane in a similar manner to melittin [22].

The perturbation of the inner membrane by peptides was investigated utilizing diSC_3_-5, a probe dependent on the membrane potential. According to the results shown in Figure 8, the peptides showed fluctuations in membrane potentials. The disruption of the cytoplasmic membrane potential led to the dissipation of the proton-motivated force, which hindered the transfer of electrons along the breathing pathway and consequently reduced the synthesis of adenosine 5-triphosphate (ATP). This reduction in ATP synthesis was crucial for the movement of bacteria that relied on flagella [23]. *E. coli* 8739 and 2340 were used to measure because of the existing flagellum. The study examined the consequences of peptides on bacterial swimming motion on low-viscosity swim plates. The peptide 14V5K demonstrated a more potent effect on the reduction of bacterial swimming diameter than melittin, whereas SS1 showed a greater capacity for membrane depolarization, as shown in Figure S7. Compared to 14V5K, the lower outer membrane binding ability and penetration effect of SS1 could be responsible for the less effective suppression of bacterial swimming motility [24]. Electrostatic attraction between AMPs and bacterial surfaces has been proposed as a crucial factor in the efficacy of AMPs. This attraction allows AMPs to reach the required threshold concentrations for membrane disruption. In conclusion, SS1 and 14V5K eliminated *E. coli* 8739 and 2340, respectively, via a membrane-targeting mechanism.

### 3.5. Antiproliferative Activity

Generally, SS1 and its analogues showed notable antiproliferative effects against both H838 and H460 lung cancer cell lines, as displayed in Table 8. However, the antiproliferative activity of L2V was better than its antimicrobial activity. This was based on the membrane-targeting mechanism. A component of the membrane surface, phosphatidylserine (PS), has a higher affinity for L2V than other peptides. In cancer cells, PS is often exposed to the outer membrane, leading to the development of a negative surface charge. This charge is associated with the lower pH in the surrounding environment. Membranolysis by AMPs and their selective mode of action in tumor cells can be attributed to the increased anionic nature of the cytoplasmic membrane of these cells. Another possibility is that L2V had similar high hydrophobicity and hydrophobic moments, which are some of the forces that are required to disrupt cell membranes. In summary, 14VL23, L2V, and SS1 showed higher selectivity against H838 and H460 cell lines, which may have the potential for further research (Table 9).

### 3.6. Haemolysis v.s. HaCat

SS1 and its analogues showed cytotoxicity mostly at high concentrations in the haemolysis assays, but the IC_50_ values of several peptides against HaCat cells were at low concentration levels. Meanwhile, the results of the salt stability assay, as shown in Table 7, revealed that SS1 lost antimicrobial activity partly in the presence of salts, but the salt stability of 14V5K was better than that of SS1. When a peptide bonded to red blood cells (RBCs), the activity of Na^+^-K^+^-ATPase on the surface of RBCs was inhibited [25]. The imbalance in intracellular potassium ion concentration led to abnormal ion exchange between the outside and inside of the membrane. In addition, the positively charged region of the peptide interfered with the transport function of anion channels in erythrocytes. This changed the osmotic pressure of the erythrocyte membrane, causing the erythrocyte to swell and lyse. However, SS1 may lose activity when potassium ions are present and may show low cytotoxicity to RBCs. Therefore, the evidence for peptides with low cytotoxicity may be explained by their poor salt displacement capacity.

## 4. Materials and Methods

### 4.1. Acquisition of Phyllomedusa tarsius Dermal Secretions

The *Phyllomedusa tarsius* frogs were procured from an industrial supplier (PeruBiotech E.I.R.L., Lima, Peru). The epidermal secretions were collected using percutaneous electrical stimulation. The skin secretions were washed away from the skin surface using deionized water and then collected in a glass beaker. Subsequently, the accumulated secretions were subjected to freezing using liquid nitrogen and then freeze-dried. Afterwards, the samples were stored at −20 °C.

### 4.2. ‘Shotgun’ Cloning of a cDNA Encoding Dermaseptin-SS1 Peptide Biosynthetic Precursor

To determine the nucleotide sequence of the precursor of Dermaseptin-SS1, 5 mg of freeze-dried skin secretion was first dissolved in 1 mL lysis/binding buffer (Dynal Biotech, Merseyside, UK). Later, the isolation process was carried out using the magnetized Dynabeads^TM^ mRNA Purification Kit (Invitrogen, Oslo, Norway) according to the rule of adenine-thymine pairing. The separated mRNA was then used as a starting point to construct the cDNA library’s first strand, employing the Clontech SMARTer^®^ RACE 5′/3′ Kit (Takara Bio, USA, Inc., CA, USA). After that, the 3′-Rapid Amplification of cDNA Ends (RACE) Polymerase Chain Reaction (PCR) was carried out by applying the nested universal primer (NUP) provided by the product as an antisense primer. Additionally, the degenerate sense primer (5′-ACTTTCYGAWTTRYAAGMCCAAABATG-3′) (where Y represents C or T; W represents A or T; R represents A or G; M represents A or C; B represents T, C, or G) was utilized. This primer was derived from a previously published nucleotide sequence of the extremely conserved signal peptide of dermaseptin peptides from *Phyllomedusa tarsius*. The molecular weights of the PCR outcomes were analysed using gel electrophoresis with an ultraviolet (UV) imaging system and purified using a HiBind^®^ DNA Mini Column (Omega Bio-Tek, USA). Then, the pure products were ligated by applying a pGEM^®^-T Easy Vector System (Promega, Southampton, UK) and chosen via white and blue screening. The isolated DNA plasmids were amplified via PCR, checked using gel electrophoresis, and purified utilizing the HiBind^®^ DNA Mini Column. Finally, thermal cycling performed the sequencing reaction utilizing a BigDye Sequencing Buffer (Applied Biosystems, Foster City, CA, USA). An ABI 3100 automatic capillary sequencer (Applied Biosystems, Foster City, CA, USA) was employed to identify the nucleotide sequences of the chosen cloned samples. The nucleotide sequence was translated to amino acid sequence and the analysis of this was undertaken by using NCBI-Protein BLAST (https://blast.ncbi.nlm.nih.gov/Blast.cgi/ (accessed on 1 August 2023)). Sequences with high identities were aligned with the novel peptide sequence by Clustal-Omega (https://www.ebi.ac.uk/Tools/msa/clustalo/ (accessed on 1 August 2023)).

### 4.3. Physicochemical Properties and Modification of SS1

The structural properties of SS1 were analysed using a variety of bioinformatics tools. PEPFOLD 3 (https://bioserv.rpbs.univ-paris-diderot.fr/services/PEP-FOLD3/ (accessed on 1 August 2023)) was used to predict the secondary structure. The physicochemical properties were acquired via Heliquest (https://heliquest.ipmc.cnrs.fr/cgi-bin/ComputParams.py (accessed on 1 August 2023)). Analogues were designed according to the requirements for increasing anti-planktonic bacterial activity and modifying physicochemical properties.

### 4.4. Synthesis and Identification of SS1 and its Analogues

According to the peptide sequence, each amino acid powder was weighed in vials, and HBTU was added as a catalyst. Peptides were then chemically synthesized via solid-phase fluorenyl methoxycarbonyl (Fmoc) chemistry in a Tribute^TM^ automatic solid-phase peptide synthesizer (Protein Technologies, Tucson, AZ, USA). Rink amide resin (MBHA resin) (Millipore Sigma, Burlington, MA, USA) was used as the medium in the synthetic procedure. MBHA resin has 100–200 mesh and 0.65 mmol/g loading. The artificial peptides were liberated from the resin due to the addition of a cleavage mixture solution consisting of 94% trifluoroacetic acid (TFA), 2% deionized water (ddH_2_O), 2% thioanisole (TIS), and 2% 1,2-ethanedithiol (EDT). The process was carried out at room temperature ranging from 120 min to 240 min, and diethyl ether was added to the peptide to precipitate. After storing at −20 °C overnight, the peptides were washed with additional diethyl ether treatments. Following the approach of lyophilization, the crude peptides were subsequently purified using reverse-phase high-performance liquid chromatography (RP–HPLC) (Phenomenex Aeris PEPTIDE 5 μm XB-C18 column, 250 mm × 21.2 mm, Macclesfield, Cheshire, UK) with a linear gradient formed by buffer A (TFA/ddH_2_O = 0.05/99.95, *v*/*v*) and buffer B (TFA/ddH_2_O/acetonitrile = 0.05/19.95/80.0, *v*/*v*/*v*) at a flow rate of 5 mL/min within 80 min. The purity of peptides was analysed using MALDI-TOF mass spectrometry (Voyager DE, Perspective Biosystem, Foster City, CA, USA). The analysis was performed in the positive detection mode employing α-cyano-4-hydroxycinnamic acid (CHCA) as the matrix. The MALDI-TOF mass spectra of peptides were obtained using mMass.

### 4.5. Secondary Structure Determinations by Circular Dichroism

As previously mentioned, the secondary structures of peptides were investigated using a JASCO-815 circular dichroism (CD) spectrometer (Jasco, Essex, UK) [26]. Briefly, peptide samples at a concentration of 100 µM were mixed in solutions of 10 mM NH_4_Ac and 50% TFE/NH_4_Ac (*v*/*v*). The samples were placed in a quartz cuvette with a thickness of 1 mm and analysed using a voltage ranging from 190 to 260 nm. The scanning speed utilized in the experiment was recorded as 200 nm per minute, while the bandwidth and data pitch were determined to be 1 nm and 0.5 nm.

### 4.6. Anti-planktonic Microorganism Activity Study

The anti-planktonic microorganism efficacy of the peptides was investigated via minimal bacterial inhibitory concentration (MIC) and minimal bactericidal concentration (MBC) assays. Eight types of microorganisms, including Gram-positive bacteria, *Staphylococcus aureus* (ATCC CRM 6538), Methicillin-resistant *Staphylococcus aureus* (NCTC 12493), and *Enterococcus faecium* (NCTC 12697), Gram-negative bacteria, *Escherichia coli* (ATCC CRM 8739, ATCC BAA 2340, ATCC 13846, ATCC BAA 2469, and ATCC BAA 2471), *Klebsiella pneumoniae* (ATCC 43816), *Pseudomonas aeruginosa* (ATCC CRM 9027) and *Acinetobacter baumannii* (ATCC BAA 747) and a yeast, *Candida albicans* (ATCC CRM 10231) were utilized to test the antimicrobial activity of peptides.

For the MIC assay, microorganisms were inoculated with peptides in a 96-well plate. Nutrient broth (NB) was applied for MRSA, *E. coli*, *K. pneumoniae*, and *P. aeruginosa*, tryptic soy broth (TSB) was applied for *S. aureus*, *E. faecium*, and *A. baumannii*, and yeast extract peptone dextrose broth (YPD-B) was used for *C. albicans.* The microorganisms were cultured (bacteria: 37 °C; yeast: 26 °C) overnight at 120 rpm and subcultured to achieve the logarithmic growth stage (5 × 10^5^ CFU/mL), as confirmed by viable cell counts. After that, 99 µL of the subculture and 1 µL of the peptide were added to the 96-well plate. The final concentration of peptides ranged from 128 to 1 µmol/L (µM) using a 2-fold dilution. In addition, four other groups were established simultaneously at the commencement of the test: a growth control group, a vehicle control group (Dimethyl Sulphoxide, DMSO), a positive control group utilizing Norfloxacin at a concentration of 2 mg/mL for bacteria, and Amphotericin B. at a concentration of 1 mg/mL for yeast, and a blank control group (sterile NB/TSB/YPD-B). The MIC values were measured after overnight incubation at 37/26 °C. The optical density (OD) values were analysed at 550 nm using a Synergy HT plate reader (BioTek, Washington, USA), and were calculated using the following equation:

(1) $$Cell\;Viability\;\left(\%\right)=[(A_{s}-A_{0})/(A_{g}-A_{0})]\times 100\%$$

where *A_s_* is the absorbance value of the sample group, *A*_0_ is the average of the absorbance values of the blank control, and *A_g_* is the average of the absorbance values of the growth control. For the MBC assay, 10 µL of the inhibited cultures on the 96-well plate were transferred onto a corresponding medium agar plate and incubated overnight at 37/26 °C to measure MBC values. The results were obtained from three independent assays.

### 4.7. Haemolysis Activity Study

To start, fresh defibrinated horse blood (TCS Biosciences Ltd., Buckingham, UK) was washed with PBS solution to obtain a clear supernatant. Then, a 4% (*v*/*v*) suspension of red blood cells was made in PBS. After this, 100 µL of the peptide solutions were incubated with 100 µL of the suspension of red blood cells in 2 mL centrifuge tubes at 37 °C for 2 h. The ultimate concentration of peptides ranged from 128 to 1 µM obtained by a 2-fold dilution. Also, two other groups were set simultaneously, including a positive control group of 1% Triton X-100 and a blank control group of phosphate-buffered saline (PBS). After incubation and centrifugation at 900× *g* for 10 min, a volume of 100 µL of the resulting supernatant from each specimen was carefully transferred to individual wells of the 96-well plate. Subsequently, the OD values were analysed using a Synergy HT plate reader (BioTek, USA) at a wavelength of 570 nm using the following equation:

(2) $$Haemolysis\;Activity\;\left(\%\right)=[(A_{s}-A_{0})/(A_{p}-A_{0})]\times 100\%$$

where *A_s_* is the absorbance value of the supernatant of the peptide group, *A*_0_ is the average absorbance value of the blank control, and *A_p_* is the average absorbance value of the positive control. The data were obtained from three separate assays.

### 4.8. Salt Sensitivity Assay

To evaluate the salt sensitivity of peptide actions against bacteria, the peptides were incubated with *S. aureus* 6538 and *E. coli* 8739 in the presence of salts (150 mM NaCl, 5 mM KCl, 6 μM NH_4_Cl, 1.5 mM MgCl_2_, 2.5 mM CaCl_2_, and 4 μM FeCl_3_) [24,27,28]. After the subculture, the peptides were incubated with the bacteria (5 × 10^5^ CFU/mL) using salts. The MICs/MBCs were then tested as described in the MIC assay, and the findings obtained in this study were obtained from three separate assays.

### 4.9. Time-Killing Kinetic Assay

The present study employed a time-dependent kinetic assay to assess the bactericidal activity of peptides against two strains of *Escherichia coli*, namely *E. coli* 8739 and *E. coli* 2340. The bacteria were subcultured, as previously described, for the purpose of conducting the MIC assay. Bacterial inoculation was performed using peptide concentrations equivalent to 4-fold MIC, 2-fold MIC, and MIC, with a bacterial concentration of 5 × 10^5^ CFU/mL. Viable cell numbers were assessed by collecting samples at various intervals (0, 5, 10, 20, 30, 60, 90, 120, and 180 min). Following incubation at a temperature of 37 °C for a duration of one night, the colonies were quantified. The findings were obtained from three separate and distinct assays.

### 4.10. LPS-Binding Assay

The lipopolysaccharide (LPS) binding affinity of the peptides was assessed using a fluorescent dye BODIPY-TR cadaverine displacement assay (BC, Sigma, USA). The trial was conducted using peptides in a 96-well black plate to achieve the expected concentrations (final concentration (c.): 0.5 μM to 32 μM) in Tris-HCl buffer (pH 7.4). The positive control was melittin, which was within the same concentration range. LPS and BC dye were mixed with Tris buffer to achieve an ultimate concentration of 25 μg/mL for LPS and 2.5 μg/mL for BC dye. After reacting for 4 h at room temperature, equal volumes of LPS solution were added to peptides in the black plate, and it was incubated at 37 °C for a duration of 1 h. The fluorescence measurements were conducted using a Synergy HT plate reader (BioTek, USA), with the excitation wavelength set at λ = 590 nm and the emission wavelength set at λ = 645 nm. The test was performed in triplicate, with each trial carried out independently.

### 4.11. Outer Membrane Assay

The study involved the implementation of an outer membrane permeability assay employing N-Phenyl-1-naphthylamine (NPN), a fluorescent dye recognized for its susceptibility to the outer membrane of Gram-negative bacteria. For the purpose of this study, *E. coli* 8739 and *E. coli* 2340 were initially introduced into an LB medium and subjected to overnight incubation at a temperature of 37 °C. Following this, the cultures were subcultured at a temperature of 37 °C a rotational speed of 120 rpm for a duration of 2 h. The cells underwent centrifugation at a speed of 2000 rpm for 10 min. The cell pellets were washed and subsequently diluted to an OD value of 0.50 at a wavelength of 600 nanometers, which corresponded to a concentration of 1 × 10^8^ CFU/mL. This dilution was achieved using a 5 mM HEPES buffer solution supplemented with 5 mM glucose, and the pH of the buffer was adjusted to 7.4. The bacterial solution was diluted to a concentration of 1 × 10^7^ CFU/mL. Subsequently, a volume of 100 μL of bacterial culture was combined with 50 μL of peptide solution in the black 96-well plate. The peptide concentrations were determined according to the MIC values obtained from assays targeting planktonic microorganisms. Growth control was established using the HEPES buffer. Subsequently, a volume of 50 μL of NPN (at an ultimate concentration of 10 μM per well) was added to the respective wells. The fluorescence was measured in real-time using a Synergy HT plate reader (BioTek, Washington, USA), with the excitation wavelength set at λ = 360 and the emission wavelength set at λ = 460 for a duration of 60 min. The experiment was conducted in triplicate, with each trial being performed autonomously.

### 4.12. Inner Membrane Assay

The analysis of membrane permeabilization in *E. coli* 8739 and *E. coli* 2340 involved quantifying the activity of *β*-galactosidase released from the bacteria into the culture medium. This was achieved using o-nitrophenol *β*-D-galactoside (ONPG) as the substrate. The bacteria were cultivated in LB medium supplemented with a 2% lactose concentration at a temperature of 37 °C for an extended period. Following this, the bacteria were subcultured at 37 °C at a rotational rate of 120 rpm for a duration of 2 h. Subsequently, the bacterial culture was subjected to centrifugation at a speed of 2000 rpm for a period of 10 min. The cell pellets were suspended to an OD value of 600 nm of 0.5, corresponding to a concentration of 1 × 10^8^ CFU/mL. The suspension was then diluted by a factor of ten using a PBS solution containing 1.5 mM ONPG at a pH of 7.4. The experimental system consisted of a mixture containing 150 μL of bacteria and 50 μL of peptides. The peptide concentrations utilized in this study were determined using the MIC values obtained from the anti-planktonic microorganism assay. The absorbance was measured at a wavelength of 460 nm at regular intervals of 5 min over a duration of 90 min. This was achieved using a Synergy HT plate reader (BioTek, USA). The temperature of the plate reader was prewarmed and set at 37 °C, which is considered optimal for enzymatic reactions. This measurement aimed to assess the permeability of peptides by monitoring the influx of ONPG into the cells, with the absorbance serving as a dynamic indicator of this process. The experiment was conducted in triplicate, with each trial being performed independently.

### 4.13. Membrane Potential Assay

The alterations in the cytoplasmic membrane potential were quantified utilizing 3,3′-Dipropylthiadicarbocyanine Iodide (diSC_3_-5) (Sigma), a fluorescent dye that was sensitive to membrane potential. This experiment was conducted using *E. coli* 8739 and *E. coli* 2340. Initially, the bacteria were introduced into the LB medium and incubated overnight at a temperature of 37 °C. Subsequently, the bacterial culture was transferred to a fresh LB medium and incubated at a temperature of 37 °C with continuous agitation at a speed of 120 rpm for a period of 2 h. Following this, the bacterial cells were separated from the liquid medium via centrifugation at a speed of 2000 rpm for a duration of 10 min. The cell pellets were rinsed using a 5 mM HEPES buffer solution containing 20 mM glucose at a pH of 7.2. Subsequently, the cell pellets were diluted to an OD value of 0.05 (equivalent to a concentration of 1 × 10^7^ CFU/mL) using a 5 mM HEPES buffer solution containing both 20 mM glucose and 0.1 M KCl at a pH of 7.2. Subsequently, a volume of 10 µL of bacterial culture was combined with 200 μL of a 20 μM disC_3_-5 solution, and the mixture was incubated at room temperature for a duration ranging from 30 min to 1 h. The permeabilization of a 100 µL sample of bacteria was initially assessed at one-minute intervals over a period of 5 min using a Synergy HT plate reader (BioTek, Washington, USA), with the excitation wavelength set at λ = 485 nm and the emission wavelength set at λ = 645 nm. Subsequently, a volume of 10 μL of peptide solution, with a final concentration ranging from 0.5 μM to 4 μM, was introduced into a 90 μL suspension of bacteria. The experimental group designated as the positive control was exposed to melittin, with a final concentration ranging from 0.5 μM to 4 μM. The fluorescence emission of peptides and positively charged groups was recorded at one-minute intervals over a duration of 30 min. The experiment was conducted in triplicate, with each trial being performed independently.

### 4.14. Swimming Motility Assay

The motility of bacterial cells was assessed using swim plates containing a low-viscosity medium (0.3% agar media, *w*/*v*) supplemented with 5 g/L tryptone and 2.5 g/L NaCl. Initially, a volume of 10 mL of molten medium was combined with a peptide solution, and the final concentration ranged from 0.5 μM to 4 μM in a six-well plate. The mixture was subsequently subjected to a drying process that lasted for a duration of 2 h. A volume of 5 µL of bacterial culture containing 5 × 10^5^ CFU/mL was introduced into the central region of the wells. The samples were then incubated at a temperature of 37 °C for a duration of 48 h. Bacterial swimming was measured using white light emitted by an InGenius 3, 3MP 12/16bit system (Syngene, London, UK), and the diameter of bacterial motility was subsequently documented. The experiment was conducted in triplicate, with each trial being performed independently.

### 4.15. Antiproliferative Activity Study

The MTT assay was employed to evaluate the antiproliferative efficacy of the peptide on human cells encompassing both cancerous and non-cancerous cell lines. The human lung cancer cell lines, namely NCI-H838 and NCI-H460, as well as the human keratinocyte HaCat cell line were procured from the American Type Culture Collection (Rockville, Md., Virginia, USA). The H838 and H460 cell lines were grown in RPMI-1640 culture medium (Invitrogen, Paisley, UK), whereas the HaCat cell line was maintained in a DMEM culture medium (Sigma, St. Louis, MO, USA) separately in a 15 mL medium containing 10% fetal bovine serum (FBS) (Sigma-Aldrich, Missouri, USA) and 1% penicillin–streptomycin (PS) (penicillin 100 units/mL and streptomycin 100 μg/mL) (Sigma-Aldrich, USA) beforehand. Subsequently, the samples were cultured at 37 °C and supplemented with 5% carbon dioxide (CO_2_).

To seed the cells on the 96-well plate, a suitable number of cells (H838, H460: 8000 cells/100 µL; HaCat: 5000 cells/100 µL) were introduced into individual wells and subjected to a 24 h incubation period. The complete growth medium was withdrawn to starve the cells, and 100 μL of FBS-free medium was maintained for 4 h. The peptide concentration ranged from 10^−4^ M to 10^−9^ M by 10-fold dilution using an FBS-free medium. Afterwards, the medium within the wells was extracted, and 100 μL of the peptide solution at varying concentrations, along with the positive control (0.1% Triton X-100), vehicle control (0.5% DMSO), growth control, and blank control, were loaded. Each concentration required three replications. The plates were incubated at 37 °C in 5% CO_2_ for 22 h. Later, a volume of 10 μL of a solution comprising 3-(4,5-dimethylthiazol-2-yl)-2,5-diphenyltetrazolium bromide (MTT) at a concentration of 5 mg/mL was introduced into the sample and incubated for an additional 2 h. Later, the OD values were assessed utilizing a Synergy HT plate reader (BioTek, Washington, USA) operating at a wavelength of 570 nm. The viability of cells was determined using the following formula:

(3) $$\mathrm{Bacteria}\;Viability\;\left(\%\right)=[(A_{s}-A_{0})/(A_{g}-A_{0})]\times 100\%$$

where *A_s_* is the absorbance of the sample group, *A*_0_ is the average absorbance value of the blank control, and *A_g_* is the average absorbance value of the growth control. The results were obtained from three separate and distinct tests.

### 4.16. Statistical Analyses

The data presented were obtained using a minimum of three replicate tests. The data were analysed using GraphPad Prism 9.0 software (GraphPad Software Inc., San Diego, CA, USA). They are displayed as the mean values ± S.E.M. The *p*-value was determined using one-way ANOVA tests, which involved comparing the mean values of the specified data. Asterisks are used to indicate significant differences (* *p* < 0.05; ** *p* < 0.01; *** *p* < 0.001; **** *p* < 0.0001).

## 5. Conclusions

In summary, Dermaseptin-SS1 was a novel peptide discovered in frog skin secretions that exhibited antimicrobial and antiproliferative effects. When the hydrophobicity was high enough, adding an appropriate amount of charge greatly augmented the antibacterial efficacy. The high levels of hydrophobicity in the peptide may have influenced the antiproliferative activity against cell lines. This modification offered novel perspectives on manipulating and advancing peptide drugs derived from natural templates in prospective studies.

## Figures and Tables

**Table 1:** The mature amino acid sequences of peptide SS1 and its analogues.

Peptide	Length	Sequence
SS1	23	ALWKSILKNAGKAALNEINQIVQ-NH_2_
L14	22	ALWKSILKNAGKA--LNEINQIVQ-NH_2_
14V	23	ALWKSILKNAGKAVLNEINQIVQ-NH_2_
14G	23	ALWKSILKNAGKAGLNEINQIVQ-NH_2_
L2V	22	ALWKSILKNVGKV--LNEINQIVQ-NH_2_
14V5K	23	ALWKKILKNAGKAVLNEINQIVQ-NH_2_
14VL23	22	ALWKSILKNAGKAVLNEINQIV-NH_2_

The amino acid with a grey and ‘--’ mark was deleted amino acid at that position. The amino acid with a green mark was changed from Alanine (A) to Valine (V). The amino acid with a yellow mark was changed from Alanine (A) to Glycine (G). The amino acid with a pink colour was changed from Serine (S) to Lysine (K).

**Table 2:** The physicochemical parameters of peptide SS1 and its analogues.

Peptide	Hydrophobicity (H)	Hydrophobic Moment (μH)	Net Charge (z)
SS1	0.405	0.406	+3
L14	0.410	0.591	+3
14V	0.445	0.445	+3
14G	0.392	0.392	+3
L2V	0.492	0.659	+3
14V5K	0.403	0.486	+4
14VL23	0.475	0.455	+3

**Table 3:** Antimicrobial activity of SS1 and its analogues against various microorganisms.

Microorganisms	MIC/MBC (μM/μM)						
	SS1	L14	14V	14G	L2V	14V5K	14VL23
*S. aureus* ATCC CRM 6538	8/8	>128	4/4	>128	>128	4/4	>128
MRSA NCTC 12493	>128	32/32	8/8	>128	4/4	4/4	>128
*E. faecalis* NCTC 12697	>128	>128	>128	>128	>128	>128	>128
*E. coli* ATCC CRM 8739	2/4	4/4	2/2	16/16	>128	2/2	8/16
*K. pneumoniae* ATCC 43816	16/16	16/16	8/8	>128	8/>128	2/2	4/4
*P. aeruginosa* ATCC CRM 9027	32/32	16/>128	16/>128	32/>128	>128	8/8	>128
*A. baumannii* ATCC BAA 747	16/16	32/>128	4/4	>128	4/4	8/8	>128
*C. albicans* ATCC CRM 10231	>128	>128	>128	>128	>128	8/8	>128

**Table 4:** HC_50_, GM, and TI values of SS1 and its analogues.

Peptide	HC_50_^a^	GM ^b^ (μM)			TI Value ^c^		
		Gram +	Gram −	Yeast	Gram +	Gram −	Yeast
SS1	256.00	80.63	11.31	256.00	3.17	22.63	1.00
L14	256.00	128.00	13.45	256.00	2.00	19.03	1.00
14V	256.00	20.16	5.66	256.00	12.70	45.23	1.00
14G	256.00	256.00	76.11	256.00	1.00	3.36	1.00
L2V	256.00	64.00	38.05	256.00	4.00	6.73	1.00
14V5K	256.00	16.00	4.00	8.00	16.00	64.00	32.00
14VL23	256.00	256.00	38.05	256.00	1.00	6.73	1.00

^a^ HC_50_ was the minimum haemolytic concentration that caused 50% haemolysis of hRBCs. ^b^ The geometric mean (GM) of peptides against microorganisms was calculated using MICs. The formula was GM_MIC_ = $\sqrt[{n}]{{MIC}_{1}\cdot {MIC}_{2}\cdots {MIC}_{n}}$ (n: the number of the MIC value of the peptide). The MICs of bacteria were obtained from Table 3 to calculate the GM value. ^c^ The therapeutic index (TI) is a ratio that compares the blood concentration at which a drug becomes toxic and the concentration at which the drug is effective. TI values were calculated as HC_50_/GM_MIC_. Larger values indicated greater cell selectivity. When no detectable antimicrobial activity was observed at 128.00 μM, a value of 256.00 μM was utilized to calculate the TI value.

**Table 5:** Antimicrobial activity of SS1 and 14V5K against antibiotic-resistant *E. coli*.

Microorganisms	MIC/MBC (μM)	
	SS1	14V5K
*E. coli* ATCC BAA 2340	4/4	2/2
*E. coli* ATCC BAA 2469	4/4	1/2
*E. coli* ATCC BAA 2471	8/8	1/2
*E. coli* NCTC 13846	8/8	4/4

**Table 6:** HC_50_, GM, and TI values of SS1 and its analogues against antibiotic-resistant *E. coli*.

Peptide	HC_50_	GM ^a^ (μM)	TI Value
SS1	256.00	5.66	45.23
14V5K	256.00	1.68	152.38

^a^ To calculate the GM value, the MICs of bacteria were taken from Table 5.

**Table 7:** MICs/MBCs (µM) of 14V5K against *S. aureus* 6538 and *E. coli* 8739 in salts.

Salts	*S. aureus* 6538	*E. coli* 8739
MIC/MBC (μM)	4/4	2/2
NaCl (150 mM)	8/8	2/2
KCl (5 mM)	4/4	2/2
MgCl_2_ (1.5 mM)	4/4	2/2
CaCl_2_ (2.5 mM)	8/8	8/8
FeCl_3_ (4 μM)	4/4	1/1
NH_4_Cl (6 μM)	4/4	1/1

**Table 8:** IC_50_ values of SS1 and analogues against tested cell lines.

Cancer Cell Line	IC_50_^a^ (μM)						
	SS1	L14	14V	14G	L2V	14V5K	14VL23
H838	7.7	64.4	7.0	48.7	2.2	8.1	5.7
H460	1.3	20.1	4.1	31.7	0.6	6.1	16.4
HaCat	61.3	135.5	33.6	71.3	13.3	41.4	71.1

^a^ The half-maximal inhibitory concentration (IC_50_) is a measurement of the effectiveness of a compound in inhibiting cell proliferation.

**Table 9:** SI values of SS1 and analogues against tested cell lines.

Cancer Cell Line	SI *						
	SS1	L14	14V	14G	L2V	14V5K	14VL23
H838	8.0	2.1	4.8	1.5	6.0	5.1	12.5
H460	47.2	6.7	8.2	2.2	22.2	6.8	4.3

* The formula is SI = IC_50 (HaCat)_/IC_50 (cancer cells)_.

## Data Availability

The Dermaseptin-SS1 (SS1) biosynthetic precursor-encoding cDNA has been deposited in the NCBI Database under the accession number: OR365763.

## Supplementary Materials

The following supporting information can be downloaded at: https://www.mdpi.com/article/10.3390/molecules28186558/s1. Table S1: Molecular weight (MW) of SS1 and analogues; Table S2: MICs/MBCs (μM) of SS1 against *S. aureus* 6538 and *E. coli* 8739 in salts; Figure S1: MALDI-TOF MS spectrum of SS1 and analogues; Figure S2: Analytical HPLC data of the purified peptides; Figure S3: The kinetic time-killing curve of SS1 in *E. coli* 8739 and 2340; Figure S4: The LPS-binding affinity of SS1; Figure S5: The outer membrane permeabilization of SS1 against *E. coli* 8739 and 2340; Figure S6: The inner membrane permeabilization of SS1 against *E. coli* 8739 and 2340; Figure S7: The membrane potential results of SS1 against *E. coli* 8739 and 2340; Figure S8: The swimming motility data of SS1 against *E. coli* 8739 and 2340; Figure S9: Predicted helical wheel plots of peptides.
