# Supplementary material for: A Novel Antimicrobial Peptide, Dermaseptin-SS1, with Anti-Proliferative Activity, Isolated from the Skin Secretion of Phyllomedusa tarsius
Source: Molecules. 2023 Sep 11;28(18):6558. doi: 10.3390/molecules28186558 (PMC10535717; doi:10.3390/molecules28186558)
Supplement: Supplementary file 1 [file molecules-28-06558-s001.zip › molecules-2566358-supplementary.pdf]

Appendix A. Supplementary data

Table S1. Molecular weight (MW) of SS1 and analogues.

Peptide	MW	Sequence
SS1	2521	ALWKSILKNAGKAALNEINQIVQ-NH_2
L14	2450	ALWKSILKNAGKALNEINQIVQ-NH_2
14V	2549	ALWKSILKNAGKAVLNEINQIVQ-NH_2
14G	2507	ALWKSILKNAGKAGLNEINQIVQ-NH_2
L2V	2506	ALWKSILKNVGKVLNEINQIVQ-NH_2
14V5K	2590	ALWKKILKNAGKAVLNEINQIVQ-NH_2
14VL23	2421	ALWKSILKNAGKAVLNEINQIV-NH_2

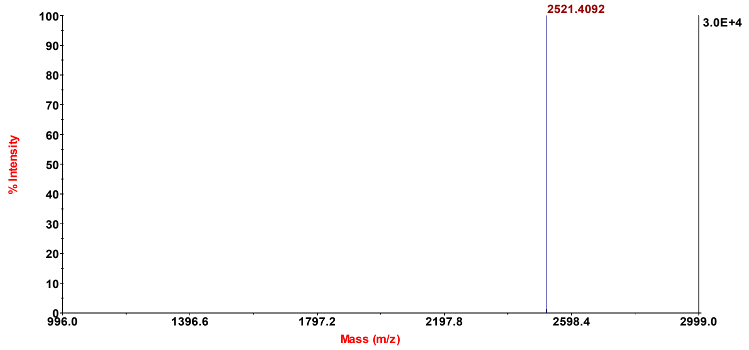

(a)

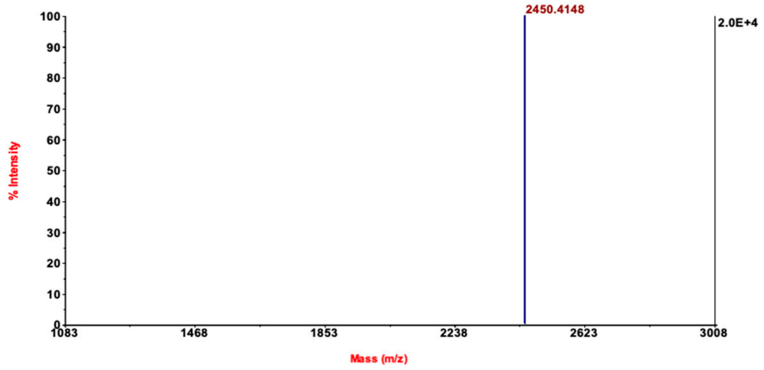

(b)

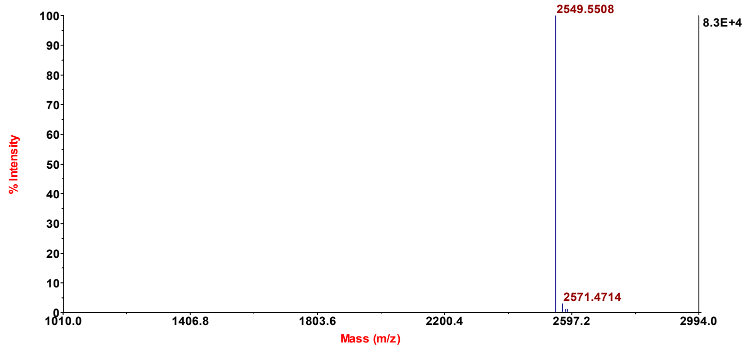

(c)

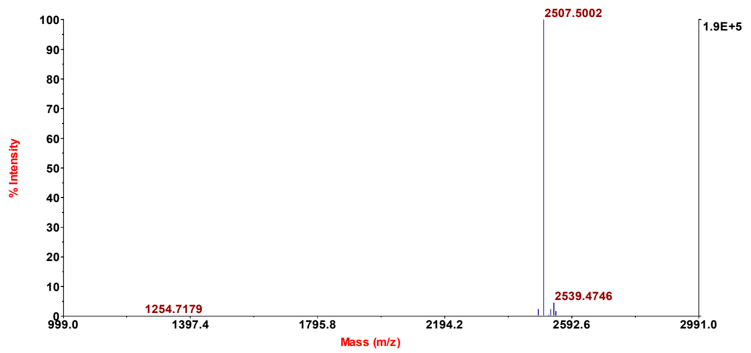

(d)

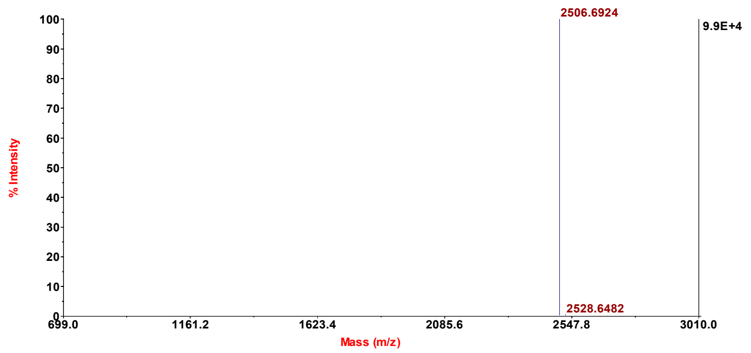

(e)

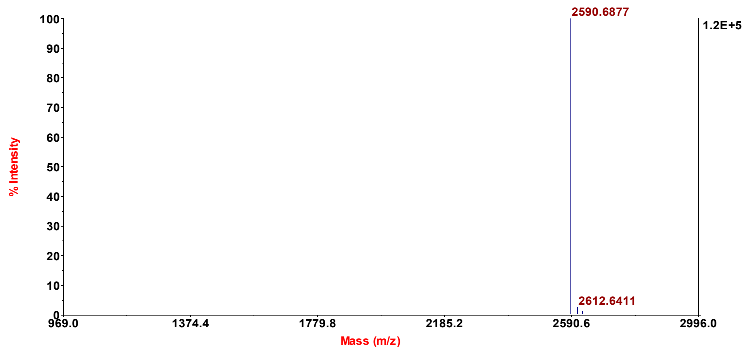

(f)

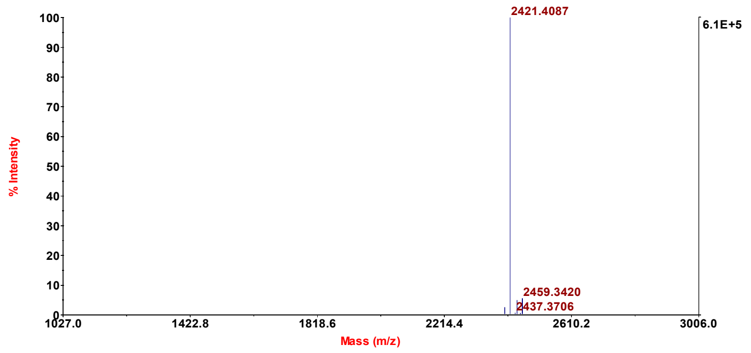

(g)

Figure S1. MALDI-TOF MS spectrum of SS1 and analogues **(a-g)**.

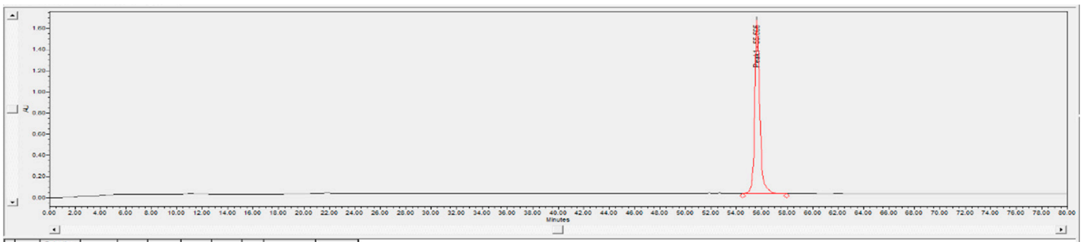

	Name	Retention Time (min)	Area (µV*sec)	% Area	Height (µV)	Int Type	Amount	Units	Peak Type	Peak Codes
1	Peak1	55.595	45415018	100.00	1636830	VV			Found	Q20

(a)

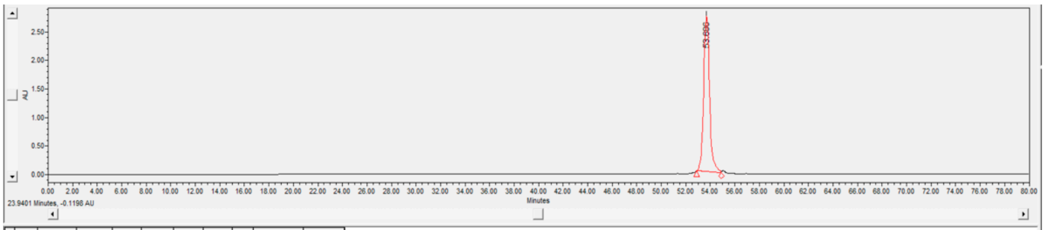

	Name	Retention Time (min)	Area (µV*sec)	% Area	Height (µV)	Int Type	Amount	Units	Peak Type	Peak Codes
1		53.686	91815696	100.00	2724003	bV			Unknown	

(b)

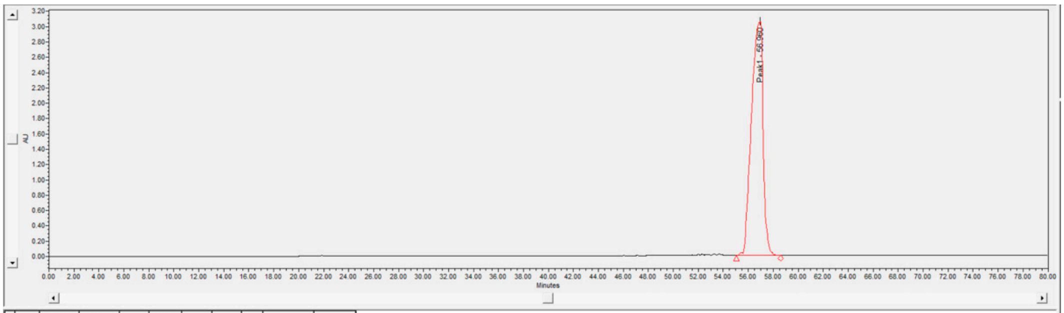

	Name	Retention Time (min)	Area (µV*sec)	% Area	Height (µV)	Int Type	Amount	Units	Peak Type	Peak Codes
1	Peak1	56.960	197100985	100.00	3050778	BV			Found	Q20

(c)

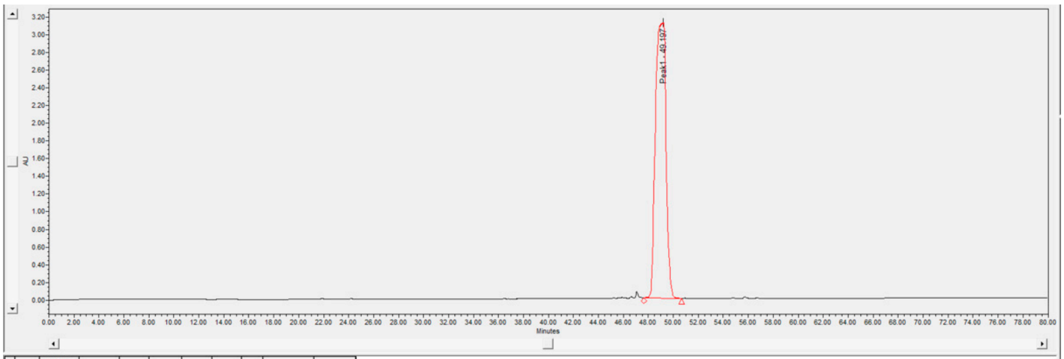

	Name	Retention Time (min)	Area (µV*sec)	% Area	Height (µV)	Int Type	Amount	Units	Peak Type	Peak Codes
1	Peak1	49.197	182991623	100.00	3109772	VB			Found	Q20

(d)

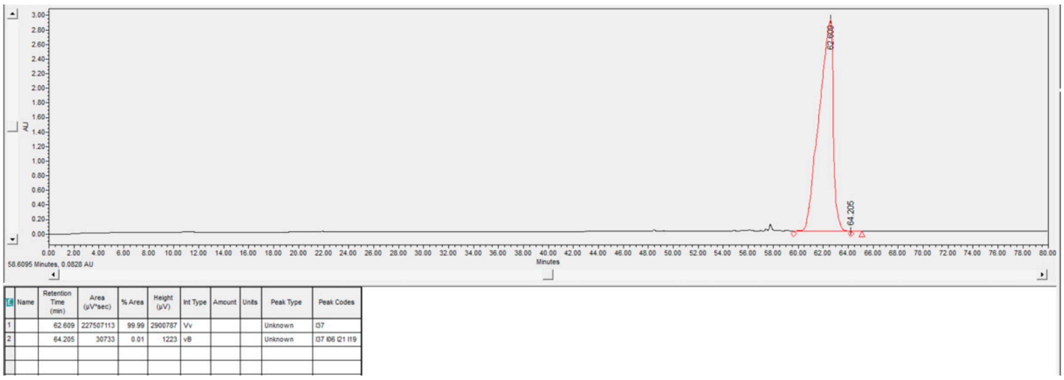

(e)

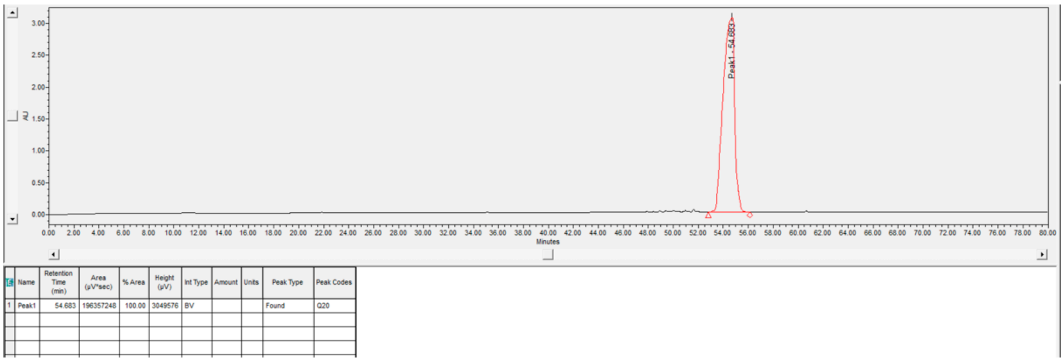

(f)

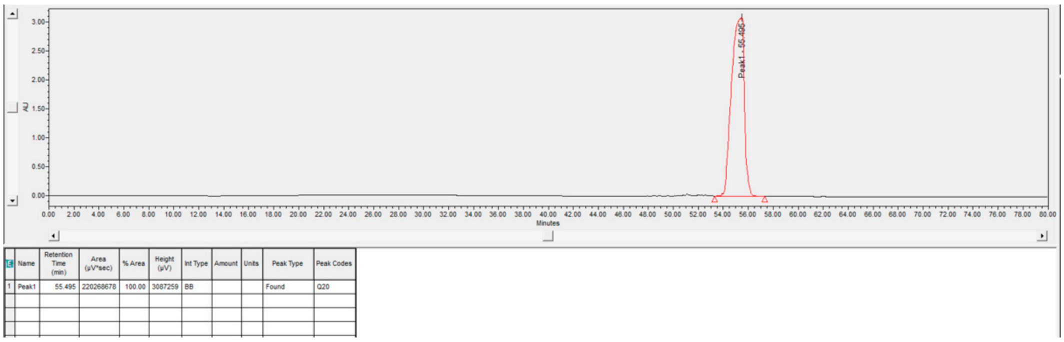

(g)

Figure S2. Analytical HPLC data of the purified peptides SS1 **(a)**, L14 **(b)**, 14V **(c)**, 14G **(d)**, L2V **(e)**, 14V5K **(f)**, 14VL23 **(g)**.

Table S2. MICs/MBCs (µM) of SS1 against *S. aureus* 6538 and *E. coli* 8739 in salts.

**Salts**	***S. aureus* 6538**	***E. coli* 8739**
MIC/MBC (µM)	8/8	2/4
NaCl (150 mM)	>128	4/4
KCl (5 mM)	>128	4/4
$MgCl_2$ (1.5 mM)	>128	16/16
$CaCl_2$ (2.5 mM)	>128	>128
$FeCl_3$ (4 µM)	>128	4/4
NH_4Cl (6 µM)	>128	2/2

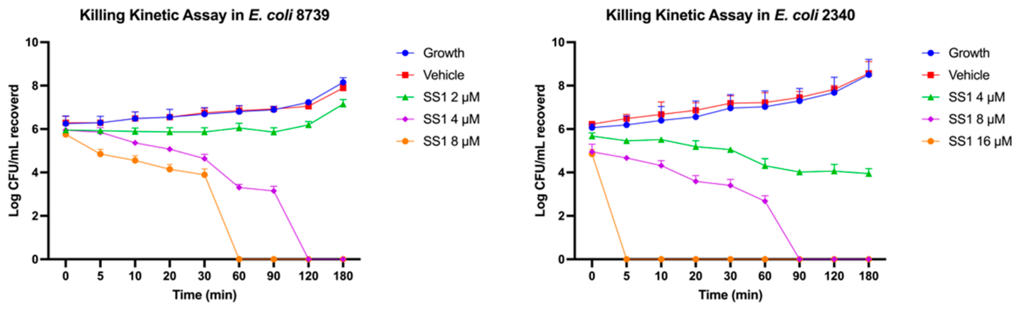

(a) **(b)**

Figure S3. The kinetic time-killing curve of SS1 in *E. coli* 8739 and 2340 **(a,b)**.

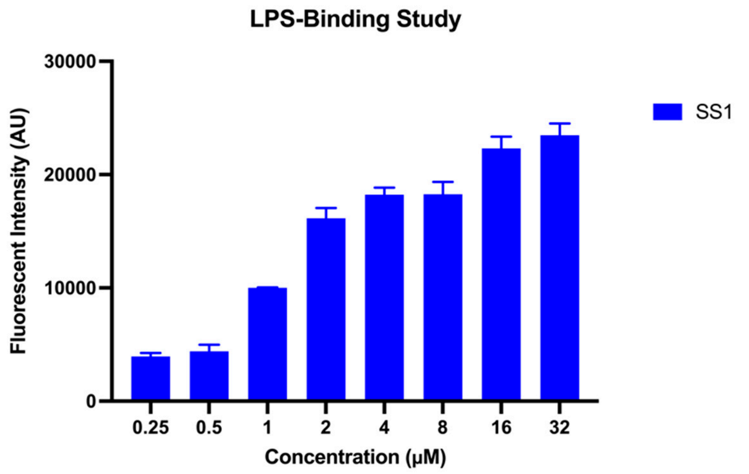

Figure S4. The LPS-binding affinity of SS1.

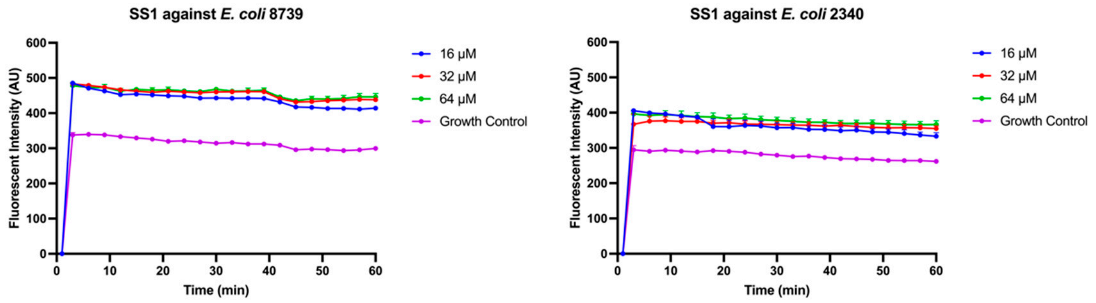

(a) **(b)**

Figure S5. The outer membrane permeabilization of SS1 against *E. coli* 8739 and 2340 **(a,b)**.

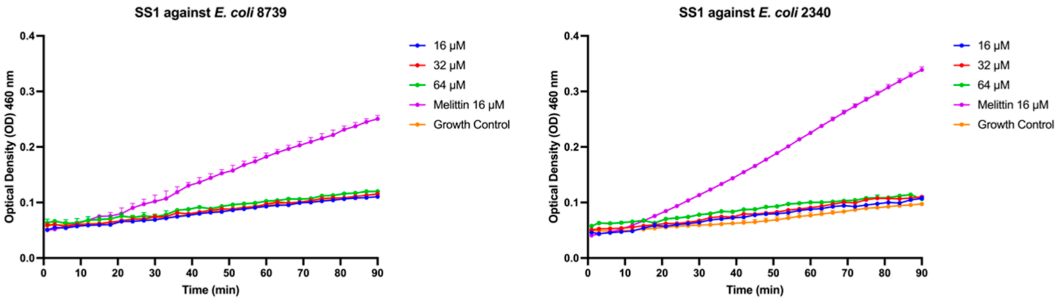

(a) **(b)**

Figure S6. The inner membrane permeabilization of SS1 against *E. coli* 8739 and 2340 **(a,b)**.

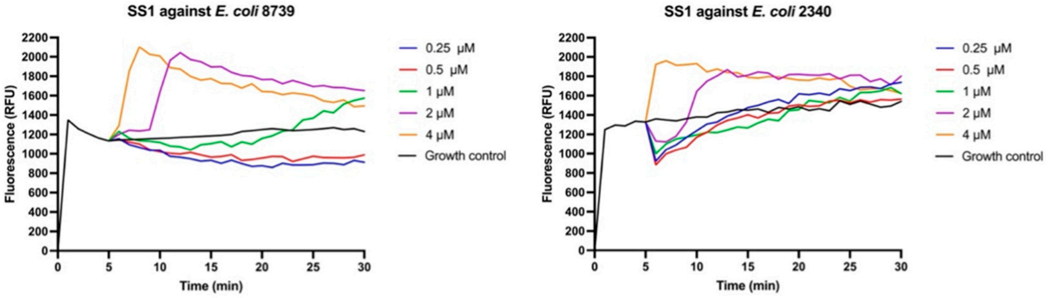

(a) **(b)**

Figure S7. The membrane potential results of SS1 against *E. coli* 8739 and 2340 **(a,b)**.

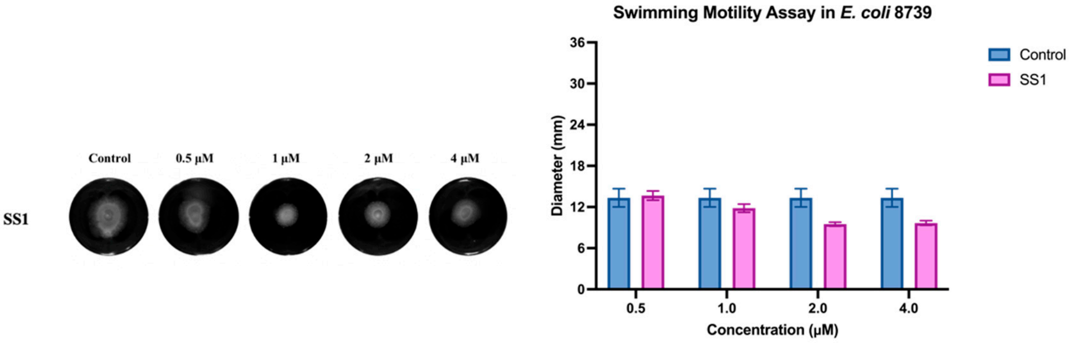

(a)

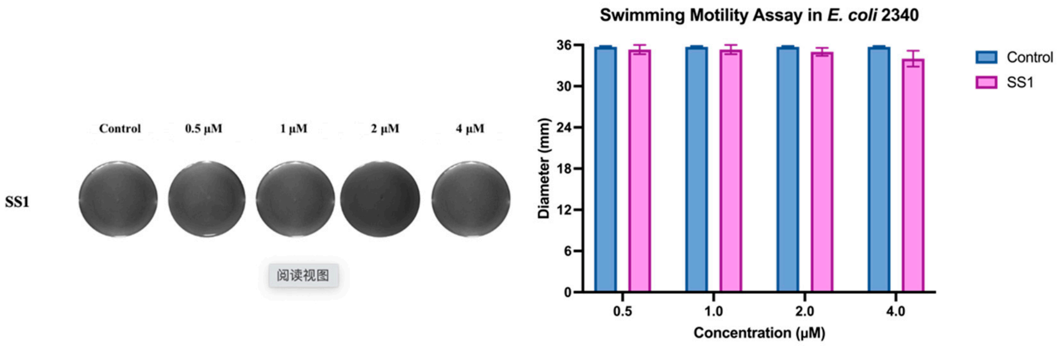

(b)

Figure S8. The swimming motility data of SS1 against *E. coli* 8739 and 2340 **(a,b)**.

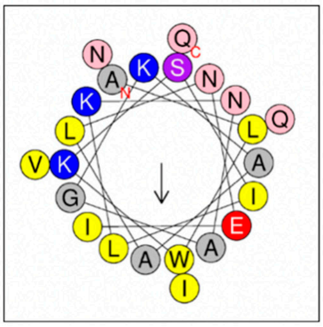

(a) SS1

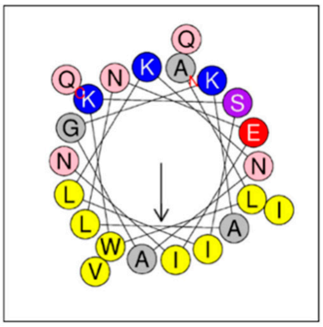

(b) L14

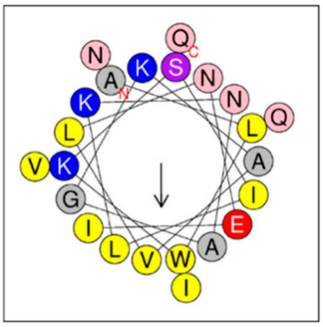

(c) 14V

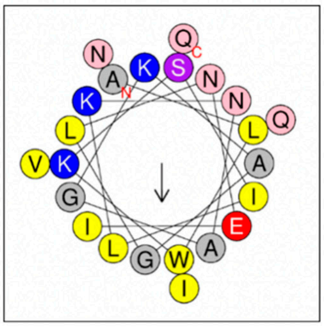

(d) 14G

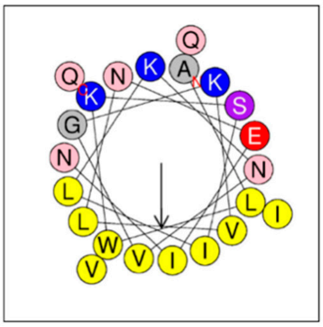

(e) L2V

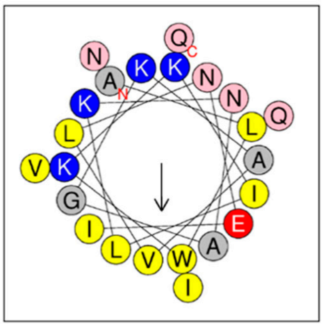

(f) 14V5K

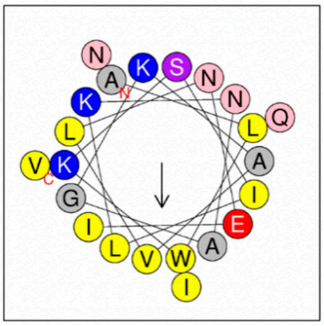

(g) 14VL23

Figure S9. Predicted helical wheel plots of peptides **(a-g)**. The direction of the hydrophobic surface was pointed by an arrow. Nonpolar residues were shown in yellow. Polar residues were shown in purple. Positively charged amino acids were shown in blue. Amino acids with negative charge were shown in red. Alanine and glycine were shown in grey. Asparagine was demonstrated in pink.
